# Randomized controlled trial of the effectiveness of an email-based alcohol intervention among university students: dismantling the assessment and feedback components

**DOI:** 10.1186/1940-0640-7-S1-A91

**Published:** 2012-10-09

**Authors:** Preben Bendtsen, Jim McCambridge, Marcus Bendtsen, Nadine Karlsson, Per Nilsen

**Affiliations:** 1 Department of Medicine and Health, Linköping University, Linköping, Sweden; 2 London School of Hygiene & Tropical Medicine, University of London, London, UK; 3 Department of Computer and Information Science, Linköping University, Sweden

## 

University students in Sweden routinely receive e-mail–based alcohol interventions sent from student health services. Earlier trials have examined the effectiveness of these interventions in simple parallel group designs. This exploratory study was undertaken in preparation for a larger trial. Using a dismantling design, we randomized 5227 students to either routine assessment and feedback (Group 1); assessment-only without feedback (Group 2); or no assessment and no feedback (Group 3). At baseline, all participants were blinded to study participation, with no contact made with Group 3. At 6-8 week follow-up, students were approached to participate in a cross-sectional alcohol study. Overall, 45% (N = 2336) of those targeted for study completed follow-up. Attrition was similar in Groups 1 and 2 (approximately 41% retained) but somewhat lower in Group 3 (52% retained). Intention-to-treat analyses among all participants, regardless of their baseline drinking status, revealed no differences between groups. Per-protocol analyses of Groups 1 and 2 among those who accepted the e-mail intervention offer (approximately 37%) and who screened positive for risky drinking (62% follow-up rate) suggested small beneficial effects on weekly consumption attributable to feedback. E-mail offer of alcohol intervention alone in an unselected population of university students was not found to be beneficial, although between-group differences in attrition prevent strong conclusions. Small benefits may follow actual uptake of e-mailed feedback intervention. The design of the main trial was positively influenced by data from this unusually large pilot study.

